# Safety and efficacy of combination of suberoylamilide hydroxyamic acid and mitomycin C in reducing pro-fibrotic changes in human corneal epithelial cells

**DOI:** 10.1038/s41598-021-83881-y

**Published:** 2021-02-23

**Authors:** Rohit Shetty, Nimisha Rajiv Kumar, Murali Subramani, Lekshmi Krishna, Ponnalagu Murugeswari, Himanshu Matalia, Pooja Khamar, Zelda V. Dadachanji, Rajiv R. Mohan, Arkasubhra Ghosh, Debashish Das

**Affiliations:** 1 Department of Cornea and Refractive Surgery, Narayana Nethralaya Eye Hospital, Bangalore, Karnataka India; 2 GROW Laboratory, Narayana Nethralaya Post Graduate Institute of Ophthalmology, Narayana Nethralaya Foundation, Narayana Nethralaya, Narayana Health City, Bommasandra, , Bangalore, Karnataka 560 099 India; 3 Stem Cell Research Lab, GROW Laboratory, Narayana Nethralaya Foundation, Narayana Nethralaya, Bangalore, Karnataka India; 4 Department of Veterinary Medicine and Surgery, University of Missouri, Columbia, MO 65211 USA; 5 Mason Eye Institute, School of Medicine, University of Missouri, Columbia, MO 65212 USA; 6 Harry S Truman Veterans’ Memorial Hospital, Columbia, MO 65201 USA

**Keywords:** Translational research, Mechanisms of disease

## Abstract

Corneal haze post refractive surgery is prevented by mitomycin c (MMC) treatment though it can lead to corneal endothelial damage, persistent epithelial defects and necrosis of cells. Suberanilohydroxamic acid (SAHA) however has been proposed to prevent corneal haze without any adverse effects. For clinical application we have investigated the short and long term outcome of cells exposed to SAHA. Human donor cornea, cultured limbal epithelial cells, corneal rims and lenticules were incubated with SAHA and MMC. The cells/tissue was then analyzed by RT-qPCR, immunofluorescence and western blot for markers of apoptosis and fibrosis. The results reveal that short term exposure of SAHA and SAHA + MMC reduced apoptosis levels and increased αSMA expression compared to those treated with MMC. Epithelial cells derived from cultured corneal rim that were incubated with the MMC, SAHA or MMC + SAHA revealed enhanced apoptosis, reduced levels of CK3/CK12, ∆NP63 and COL4A compared to other treatments. In SAHA treated lenticules TGFβ induced fibrosis was reduced. The results imply that MMC treatment for corneal haze has both short term and long term adverse effects on cells and the cellular properties. However, a combinatorial treatment of SAHA + MMC prevents expression of corneal fibrotic markers without causing any adverse effect on cellular properties.

## Introduction

There has been an ever increase in patients with refractive errors or ametropia world wide^1^. Anatomical correction of the refractive errors with surgical techniques using argon fluoride excimer laser is a widely accepted treatment modality^2^. Photorefractive keratectomy (PRK), laser-assisted in situ keratomileusis (LASIK), laser-assisted subepithelial keratomileusis (LASEK). The outcome of the refractive correction with all of these procedures are comparable for moderate myopia^3^. PRK is considered as the safe procedure involving surface ablation procedure. As a consequence of PRK procedure, epithelial wound healing is initiated on the corneal surface with epithelial cell proliferation, migration, fibroblast differentiation and hemidesmosome formation^4^. Post-surgery sub-epithelial fibrotic changes, transformation of keratocytes to myofibroblasts and deposition of aberrant collagen and extracellular matrix components results in development of corneal haze^5^. Corneal myofibroblasts generated from keratocytes activated by inflammatory and pro-fibrotic factors are the primary cause of haze formation^6^. Transforming growth factor beta 1 (TGFβ1) plays a crucial role in regulating corneal haze formation by modulating cell proliferation and differentiation^7^. Agents limiting keratocytes proliferation and differentiation to myofibroblast can prevent corneal haze formation post PRK.

Mitomycin C derived from Streptomyces species has anti-neoplastic and antibiotic functions exerted by its DNA alkylating property during cell division. It has been approved to be used for preventing haze on corneal surface by inducing apoptosis of keratocytes and stromal cells^8^. It has been elucidated that conditioned medium from MMC treated corneal epithelial cells lead to senescence of corneal epithelial cells and secreted senescence associated secreted proteins that suppressed the collagen deposition implicating a definitive role of corneal epithelial cells in preventing haze in the presence of MMC^9^. It has been shown that prophylactic usage of MMC has more beneficial effects rather than therapeutic effects^10^. The widely accepted prophylactic usage of MMC in ophthalmic clinics for PRK and LASEK are supported by the fact that MMC marginally reduces stromal keratocytes that can be repopulated within a year, none or minimal decrease in endothelial cell density^11–13^. However, since several animal studies have revealed decrease in endothelial cell density, further studies investigating the physiological functions are warranted^14^. The dosage and duration of MMC exposure is critical as has been reported in several studies^10^. The debilitating effects of topical application of MMC can be extended persistent epithelial defects, limbal and scleral necrosis, corneal endothelial damage and corneal perforation^15^. In an effort to explore other agents with similar functions as MMC, potential of SAHA has been evaluated.

Studies have reported that histone deacetylase inhibitors are shown to reduce TGFβ1 induced myofibroblast formation along with fibrotic changes using in-vitro model^16^. An analogue of Trichostatin A, vorinostat (Suberoylamilide Hydroxyamic Acid-SAHA) has been approved by United States Food and Drug Administration for medical use in cutaneous T-cell lymphoma^17^. In vitro as well as in vivo animal studies have conclusively demonstrated that SAHA can reduce corneal haze and also short and long term damage to corneal endothelium^18–20^. Studies of have shown SAHA to be less toxic at their efficacious dosage compared to MMC^20^. Therefore, it is imperative to establish the individual effects of SAHA on human eyes as well as its combinatorial treatment with MMC.

However, there are no safety and efficacy studies of a combinatorial treatment of SAHA and MMC on human tissues. In order to be able to investigate the applicability of the treatment for clinical trials a targeted study using human samples is warranted. Therefore, our study investigates the effect of SAHA with/without MMC in in vitro primary culture systems as a step towards understanding the safety and efficacy of the combinatorial treatment to prevent corneal haze post PRK.

## Results

### Concentration of SAHA and MMC based cell viability

**Figure 1:**
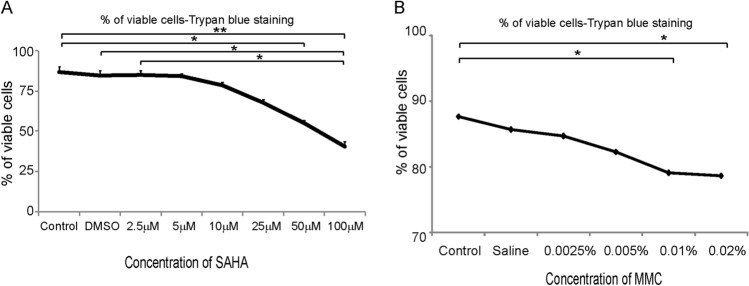
Dose dependent effect of SAHA and MMC on cell viability. Trypan blue cell viability assay of cultured day 14 differentiated human limbal epithelial cells to corneal epithelial cells treated with different concentrations of SAHA (**A**) and MMC (**B**) (n = 4). Statistical significance based on one way ANOVA test denoted by *p ≤ 0.05, **p ≤ 0.01, ***p ≤ 0.001, was calculated in presence of SAHA and MMC in comparison to untreated cultured cells by using statistical software GraphPad PRISM Ver 6.01.

Cultured corneal epithelial cells were treated with different concentrations of SAHA dissolved in DMSO or MMC dissolved in saline and incubated for 24 h. The cell viability was performed with trypan blue dye exclusion analysis. The results revealed that at concentrations higher than 10 μM there was a decrease in the percentage of viable cells in SAHA (Fig. 1A), whereas at concentrations higher than 0.005% there was a decrease in percentage of viable cells for MMC (Fig. 1B). Hence, for all our experiments we have used SAHA at a concentration of 5 μM. There was a significant decrease in the percentage of viable cells at 50 μM (p = 0.0352) and 100 μM (p = 0.0042) compared to untreated controls. Incubation with MMC showed significant decrease in viability at concentrations of 0.01% (p = 0.046) and 0.02% (p = 0.037) compared to controls. Hence, we have used 0.005% MMC and 5 μM of SAHA in this study for treating cells.

### Effect of SAHA and MMC on corneal explants

**Figure 2:**
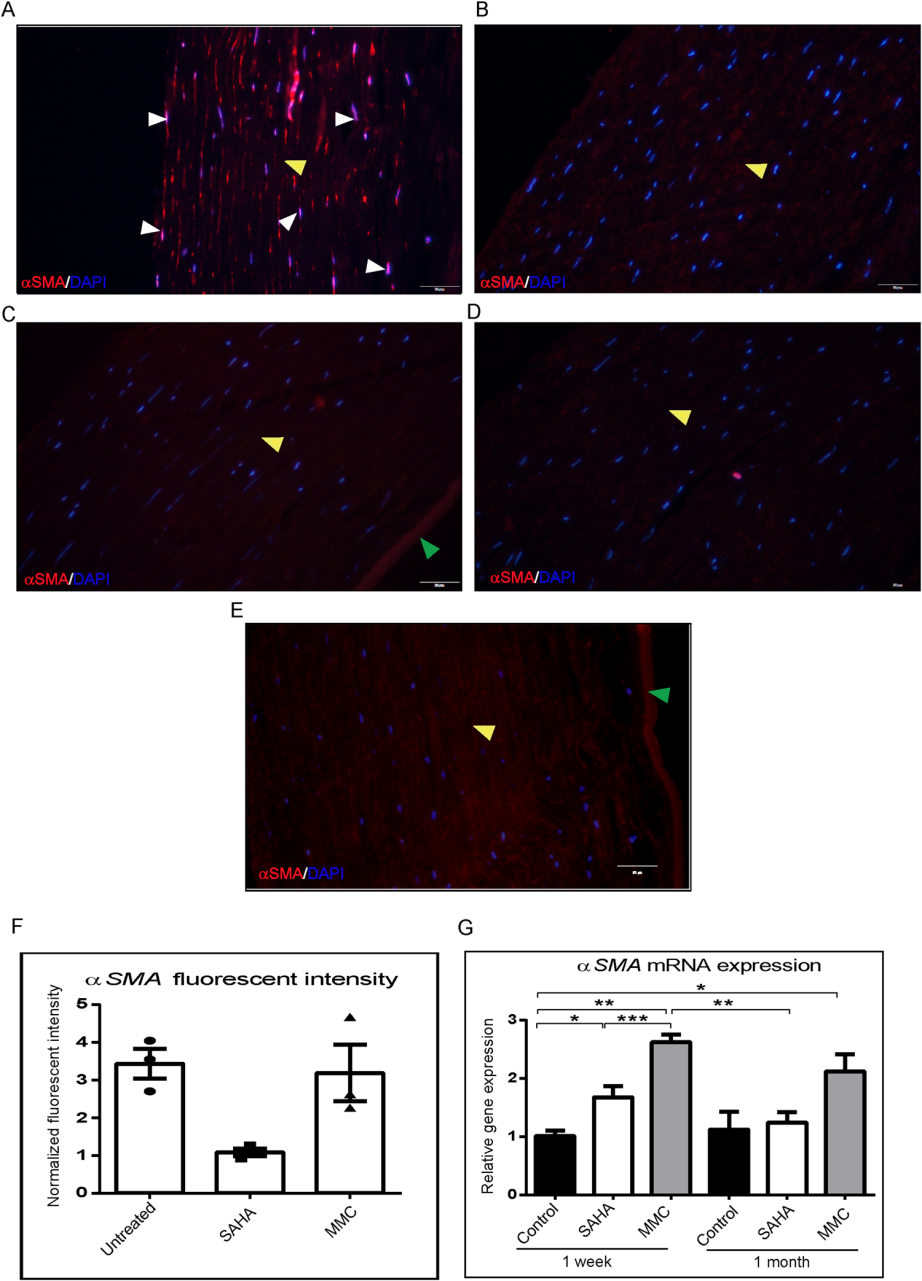
Effect of MMC and SAHA on cornea. Donor corneas were incubated for 1 month post surgery with MMC and SAHA. Representative immunofluorescence images of αSMA (red) and DAPI (blue) staining after MMC (**A**) and SAHA (**B**) treatment. Negative control for immunofluorescence images are corneas treated with MMC (**C**) and SAHA (**D**) and stained with secondary antibody only. Representative image of cornea cultured for 1 month without SAHA or MMC treatment and stained for αSMA (red) and DAPI (blue) (**E**). The white arrow heads show the αSMA positive staining, the yellow arrow head shows the stromal region and the green arrow depicts the endothelial layer of the cornea. mRNA expression of corneas (n = 4) treated with MMC (0.005%) and SAHA (5 µM) for 1 week and 1 month for αSMA levels (**G**). The mRNA levels of the gene of interest were normalized with the expression levels of β-actin gene. Mann- Whitney test for independent samples were run to get statistical significance. Images were quantified using Image J 1.48 version software (http://imagej.nih.gov/ij/) and statistical analysis performed using statistical software GraphPad PRISM Ver 6.01.

**Figure 3:**
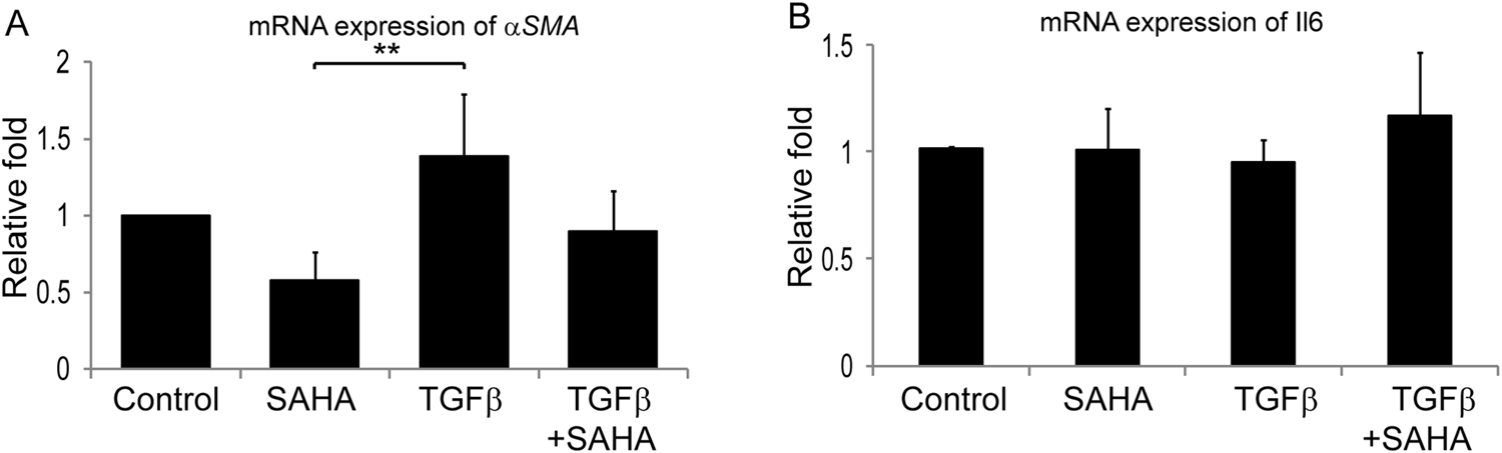
Effect of SAHA and TGFβ on cultured lenticules. Relative mRNA expression of αSMA (**A**) and IL6 (**B**) in cultured lenticules (n = 4). Obtained after SMILE surgery that were treated with SAHA (5 µM), TGFβ (10 ng/ml), SAHA (5 µM) + TGFβ (10 ng/ml) for 48hrs. Mann- Whitney test for independent samples were run to get statistical significance (p = 0.018) by using statistical software GraphPad PRISM Ver 6.01.

Corneal buttons were incubated with SAHA and MMC to evaluate their effects on expression of pro-fibrotic marker αSMA. The results revealed that MMC upregulated αSMA expression both at protein (Fig. 2A–F) as well as mRNA levels (Fig. 2G). Corneal buttons incubated with MMC revealed significant increase of αSMA mRNA levels both in 1 week (p = 0.009) as well as 1 month (p = 0.024) incubation compared to 1 week control corneal buttons. There was observed a significant reduction in the αSMA mRNA levels in corneal buttons incubated with SAHA for 1 week (p = 0.0002) and 1 month (p = 0.001) compared to those incubated with MMC for 1 week (Fig. 2G). Excised corneal lenticules were treated with SAHA, TGFβ individually as well as in combination. The mRNA expression revealed significant decrease in αSMA levels (p = 0.0022) by SAHA treatment with or without pro-fibrotic stimulation (Fig. 3A). However, no difference was detected in the mRNA levels of IL6 (Fig. 3B).

### Status of apoptosis levels in presence of SAHA and MMC

**Figure 4:**
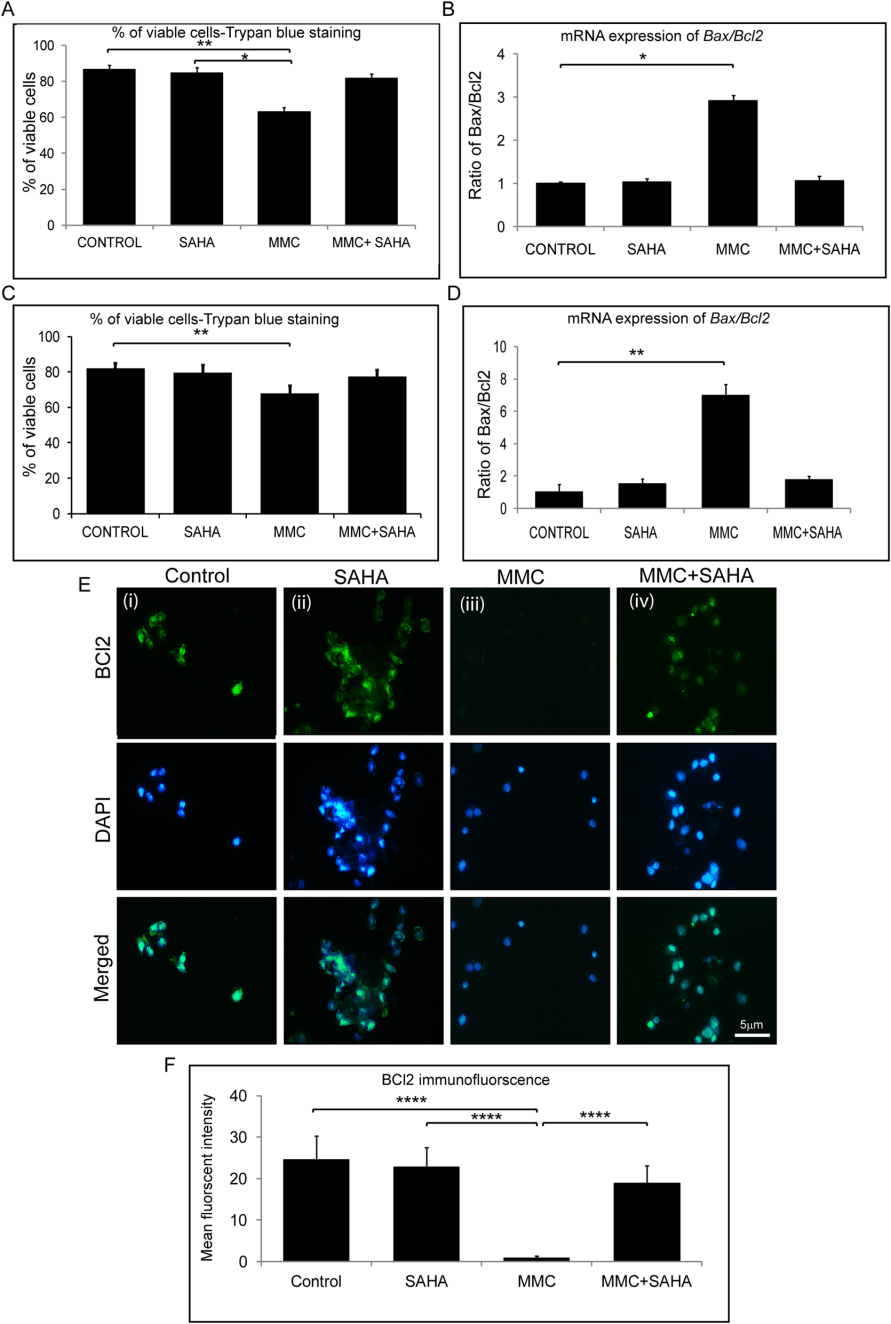
Effect of MMC and SAHA on cell apoptosis levels. Trypan blue cell viability assay results of study group 1 cultures (**A**) and cells from study group 2 (**C**) are represented graphically (n = 4). Ratio of relative mRNA expression of Bax and Bcl2 in treated cells of study group 1 (**B**) and study group 2 (**D**) are depicted (n = 4). Representative immunofluorescence staining images with BCL2 (green) and DAPI (blue) in cells obtained from study group 2 (**E**). For the immunofluorescence staining experiments were conducted (n = 3). In total 200–220 cells were analyzed per treatment group and represented graphically showing the mean fluorescent intensity of the images (**F**). Statistical significance based on one way ANOVA test denoted by *p ≤ 0.05, **p ≤ 0.01, ****p ≤ 0.0001 was calculated in presence of SAHA and MMC in comparison to untreated cultured cells. Images were quantified using Image J 1.48 version software (http://imagej.nih.gov/ij/) and statistical analysis performed using statistical software GraphPad PRISM Ver 6.01.

Cultured limbal epithelial cells were treated with SAHA and MMC alone or in combination (study group 1). The cell viability was ascertained using Trypan blue vital staining. The percentage of viable cells was similar in control cultures and those that were exposed to SAHA and MMC + SAHA. However, there was a significant decrease in the percentage of viable cells in cultures treated with MMC compared to untreated cultures (p = 0.0034) and those incubated with SAHA (p = 0.0106) (Fig. 4A). Furthermore, to determine the effect of treatment on the human eye, cadaveric corneal rims were incubated with SAHA and MMC alone or in combination (study group 2). Cell viability assay showed results similar to those obtained by incubating the reagents in cultured limbal epithelial cells. Corneal rims incubated with SAHA and MMC + SAHA did not show any difference compared to the untreated corneal rims. However, there was a significant decrease in the percentage of viable cells in rims incubated with MMC alone (p = 0.0038) compared to untreated rims (Fig. 4C). Gene expression analysis revealed significant upregulation of the ratio of Bax and Bcl2 levels in study group 1 (p = 0.028) as well as study group 2 (p = 0.0022) exposed to MMC compared to control cultures, respectively (Fig. 4B,D). There was no significant difference in the ratio of the mRNA levels of Bax and Bcl2 in cells obtained from cultures and corneal rims treated with SAHA and MMC + SAHA in comparison to untreated cultures. Furthermore, corneal rims of study group 2were stained for BCL2 protein expression. The results revealed a significant decrease in the mean fluorescent intensity of BCL2 positive cells obtained from corneal rims exposed to MMC compared to those in controls, SAHA and MMC + SAHA (p < 0.0001) (Fig. 4E,F).

### Status of fibrotic gene expression

**Figure 5:**
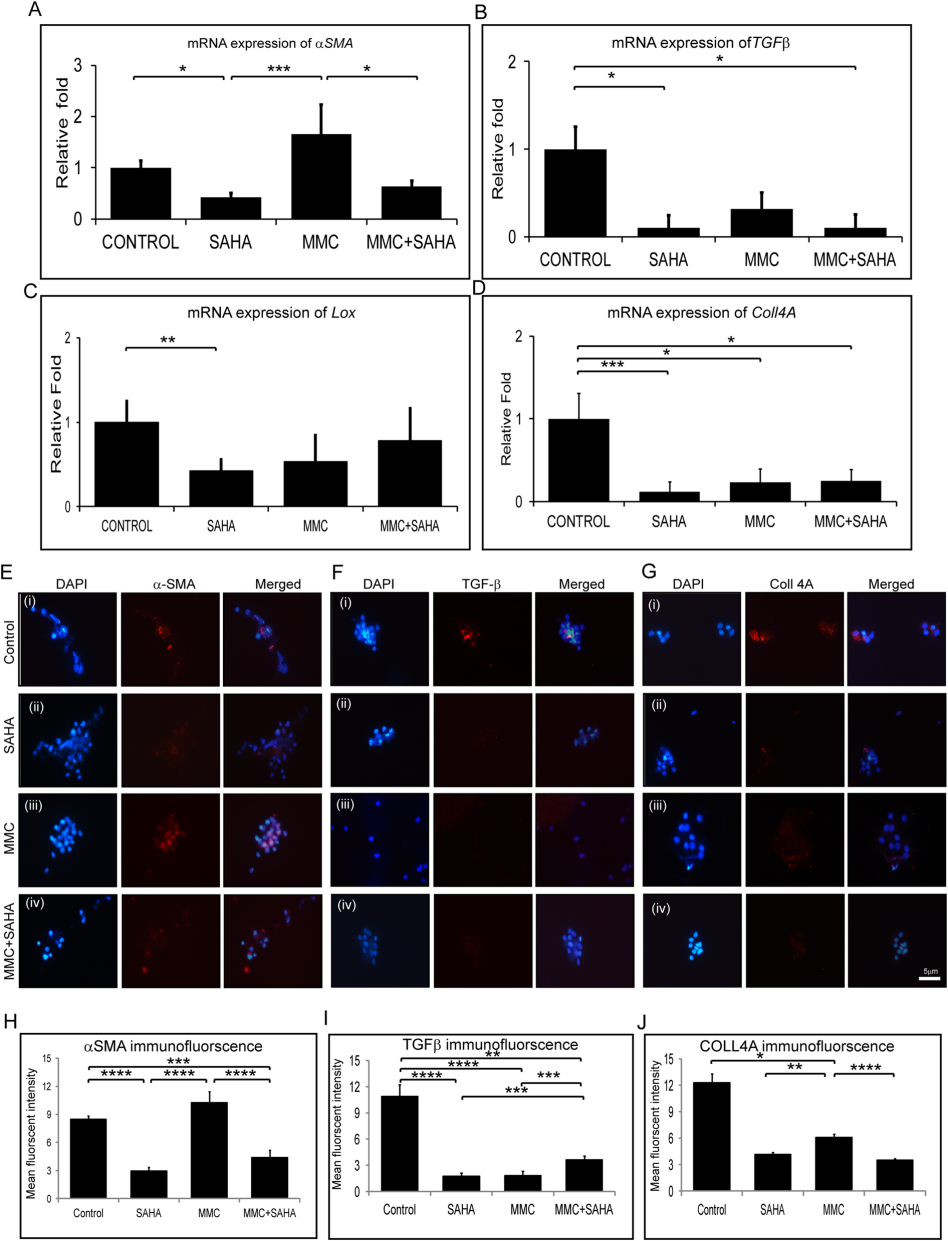
Effect of MMC and SAHA on fibrotic markers. Relative mRNA expression levels of αSMA (**A**), Tgfβ (**B**), Lox (**C**) and Coll4A (**D**) in cells obtained from study group 2 and control (n = 4). Representative immunofluorescence images with αSMA (red) (**E**), TGFβ (red) (**F**), COLL4A (red) (**G**) and DAPI (blue) in cells obtained from corneal rims of study group 2 (n = 4). Immunofluorescence experiments were conducted in triplicate and the mean fluorescent intensity was calculated for 200–220 cells and depicted graphically stained with αSMA (**H**), TGFβ (**I**), COLL4A (**J**). Statistical significance based on one way ANOVA test denoted by *p ≤ 0.05, **p ≤ 0.01, ***p ≤ 0.001, ****p ≤ 0.0001 was calculated in presence of SAHA and MMC in comparison to untreated cultured cells. Images were quantified using Image J 1.48 version software (http://imagej.nih.gov/ij/) and statistical analysis performed using statistical software GraphPad PRISM Ver 6.01.

Corneal rims from study group 2 were analyzed for expression of fibrotic markers. Corneal rims incubated with SAHA (p = 0.0004) and MMC + SAHA (p = 0.0452) showed significant decrease in the mRNA levels of α*SMA* compared to those incubated with MMC. The rims treated with MMC showed elevated expression of αSMA compared to controls. SAHA incubated corneal rims showed significant (p = 0.0452) decrease in the mRNA levels of α*SMA* compared to controls (Fig. 5A). There was a significant decrease in the mRNA levels of *TGF*β in the presence of SAHA (p = 0.0255), MMC and MMC + SAHA (p = 0.0138) compared to control rims (Fig. 5B). Similarly, the mRNA levels of *Lox* and *Coll4A* showed decreased mRNA expression levels in cells of study group 2 compared to controls (Fig. 5C,D). Significant reduction in the mRNA levels of *Lox* was noted in rims incubated with SAHA (p = 0.0022) compared to controls (Fig. 5C). Similarly *Coll4A* mRNA levels were significantly reduced in rims incubated with SAHA (p = 0.0004), MMC (p = 0.0453) and MMC + SAHA (p = 0.0453) compared to controls (Fig. 5D). Furthermore, immunofluorescence staining was performed to corroborate the results obtained by mRNA analysis using samples of study group 2. Quantification of the mean fluorescent intensity of the images revealed that cells obtained from corneal rims incubated with SAHA (p < 0.0001) and MMC + SAHA (p = 0.0002) showed significant lower expression of αSMA staining positivity compared to controls. However, cells obtained from corneal rims treated with MMC showed no change in the levels of αSMA with respect to control. The αSMA positivity was significantly high in cells obtained from rims incubated with MMC compared to those with SAHA (p < 0.0001) and MMC + SAHA (p < 0.0001) (Fig. 5E,H) Cells obtained from corneal rims incubated with SAHA (p < 0.0001; p < 0.0001), MMC (p < 0.0001; p = 0.0114) and MMC + SAHA (p = 0.0034; p < 0.0001) showed significantly lower mean fluorescent intensity of TGFβ and COLL4A levels compared to controls (Fig. 5F,G,I,J). Additionally, it was noted that TGFβ mean fluorescent intensity was significantly lower in SAHA (p = 0.0002) and MMC (p = 0.0003) incubated rims compared to those incubated with SAHA + MMC (Fig. 5I). Similar results were obtained in the levels of COLL4A mean fluorescent intensity levels. Significant reduction was observed in COLL4A levels in rims incubated with SAHA (p = 0.0057) and MMC + SAHA (p < 0.0001) compared to those incubated with MMC alone (Fig. 5J). The results show that SAHA reduced αSMA expression levels whereas MMC promoted the expression of αSMA.

Analysis of gene expression levels of study group 1 samples revealed that MMC + SAHA treatment decreased the expression of fibrotic markers compared to control as well as those incubated with MMC + SAHA. Cultures treated with MMC showed upregulation in mRNA levels of α*SMA* (p = 0.0453), *TGF*β, *Coll4A* and *Lox* (p = 0.0022) compared to the controls (Supplementary Fig. S2A–D). In the presence of SAHA, the MMC induced upregulation of mRNA levels was significantly reduced. A significant reduction of α*SMA* (p = 0.0004), *TGF*β p = 0.0022), *Lox* and *Coll4A* (p = 0.0022) mRNA levels was found in MMC + SAHA treated cultures compared to those incubated with MMC alone (Supplementary Fig. S2A–D). However, in the presence of MMC, SAHA as well as MMC + SAHA the mRNA levels of proliferative marker *Ki67* and *Cyclin D1* were reduced compared to control mRNA levels (Supplementary Fig. S2E). A significant decrease in the mRNA levels of Ki67 (p = 0.0022) and Cyclin D1 (p = 0.0022) was noted in cells incubated with MMC + SAHA compared to controls. A decrease in the mRNA levels of *Coll3A1*, *Decorin* and *Fibronectin 1* was noted in samples of study group 1 compared to controls. A significant decrease in mRNA levels of *Coll3A1* (p = 0.0022) and *Decorin* (p = 0.0022) was observed in cells treated with SAHA compared to controls. A significant decrease in the levels of *Fibronectin* (p = 0.0022) was observed in cells treated with MMC compared to controls (Supplementary Fig. S2F).

### Regulation of MDR genes in the presence of SAHA + MMC

**Figure 6:**
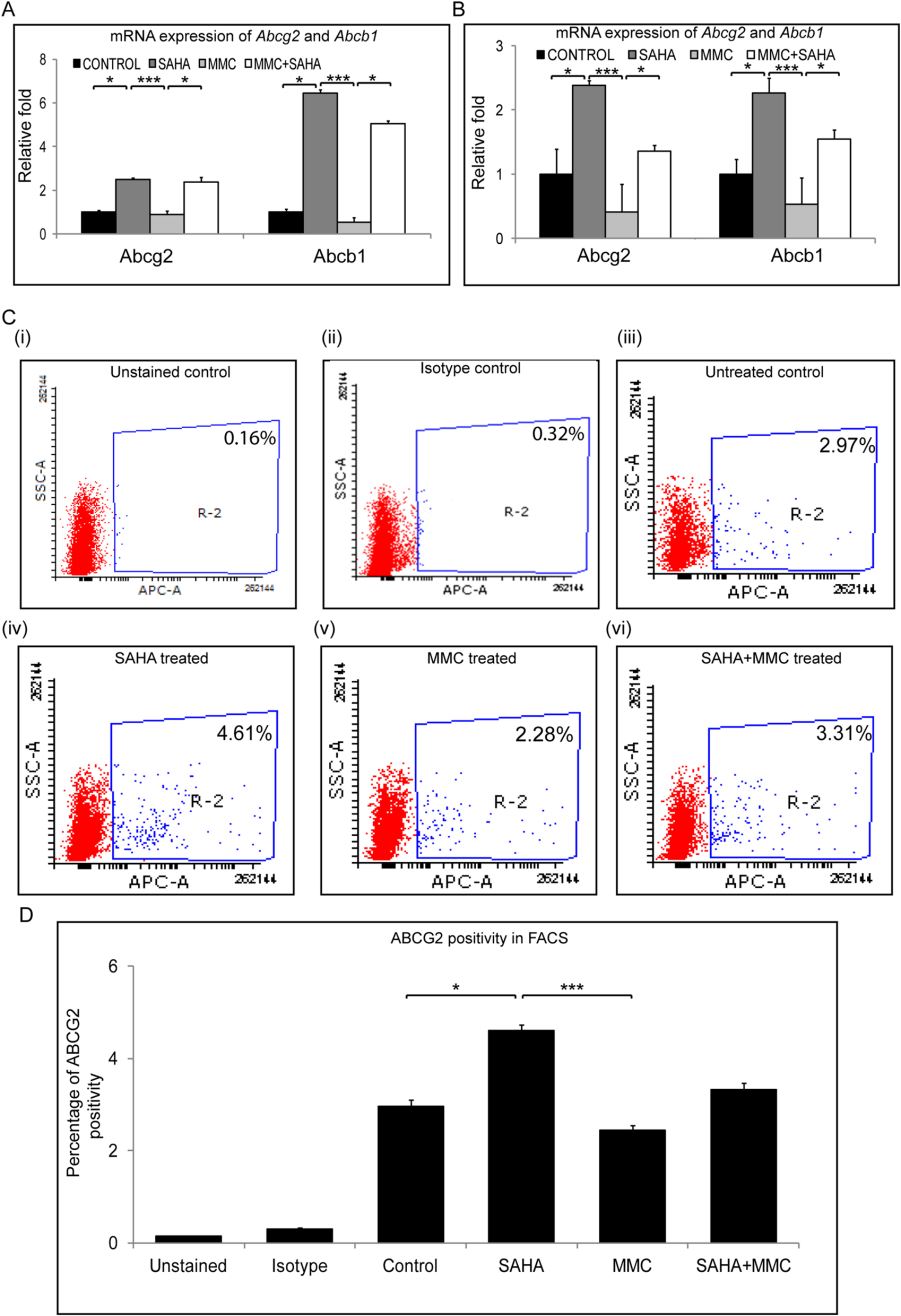
Effect of MMC and SAHA on MDR proteins. Relative mRNA expression levels of Abcg2 and Abcb1 in cells of study group 1 (**A**) and study group 2 (**B**) (n = 5). Representative FACS plots showing the effect of treatment on cells of study group 1 on ABCG2 levels (**C** (i-vi)) (n = 4). Graphical representation of percentage of ABCG2 positivity (**D**) Statistical significance based on one way ANOVA test denoted by *p ≤ 0.05, ***p ≤ 0.001 was calculated using statistical software GraphPad PRISM Ver 6.01.

In an attempt to understand the underlying mechanism for these effects of SAHA and MMC treatments, we analysed the gene expression levels of multidrug resistance (MDR) proteins. Cells obtained from study group 1 were analysed for mRNA expression of MDR proteins. In the presence of SAHA, a significant upregulation of Abcg2 (p = 0.0453) and Abcb1 (p = 0.0453) was observed compared to untreated day 14 differentiated limbal epithelial cultures. But no change was observed in expression levels of Abcg2 and Abcb1 in MMC treated cultures compared to untreated cultures. A significant decrease was found in mRNA levels of Abcg2 (p = 0.0004) and Abcb1 (p = 0.0004) in cells incubated with MMC compared to those incubated with SAHA alone. Contrarily, a significant increase in the mRNA levels of Abcg2 (p = 0.0453) and Abcb1 (p = 0.0453) in cells treated with MMC + SAHA compared to MMC (Fig. 6A). A similar result was obtained in the mRNA levels of cells obtained from the study group 2. Corneal rims incubated with SAHA showed significantly elevated mRNA levels of Abcg2 (p = 0.0453) and Abcb1 (p = 0.0453) compared to untreated controls. In the presence of MMC, the mRNA of cells from corneal rims revealed reduced levels of Abcg2 and Abcb1 compared to untreated controls. Corneal rims incubated in MMC + SAHA show significantly elevated gene expression of Abcg2 (p = 0.0453) and Abcb1 (p = 0.0453) compared to those treated with MMC alone. There was also a significant upregulation of Abcg2 (p = 0.0004) and Abcb1 (p = 0.0004) mRNA in rims treated with SAHA compared to those with MMC (Fig. 6B). Flow cytometry analysis for ABCG2 positive population in study group 1 samples revealed increase in the percentage of positive population in SAHA (p = 0.0310) compared to control. The ABCG2 positivity was significantly high in SAHA treated cultures compared to those incubated with MMC (p = 0.0006) (Fig. 6C,D).

### Differentiation status of corneal limbal epithelial cells after treatment

**Figure 7:**
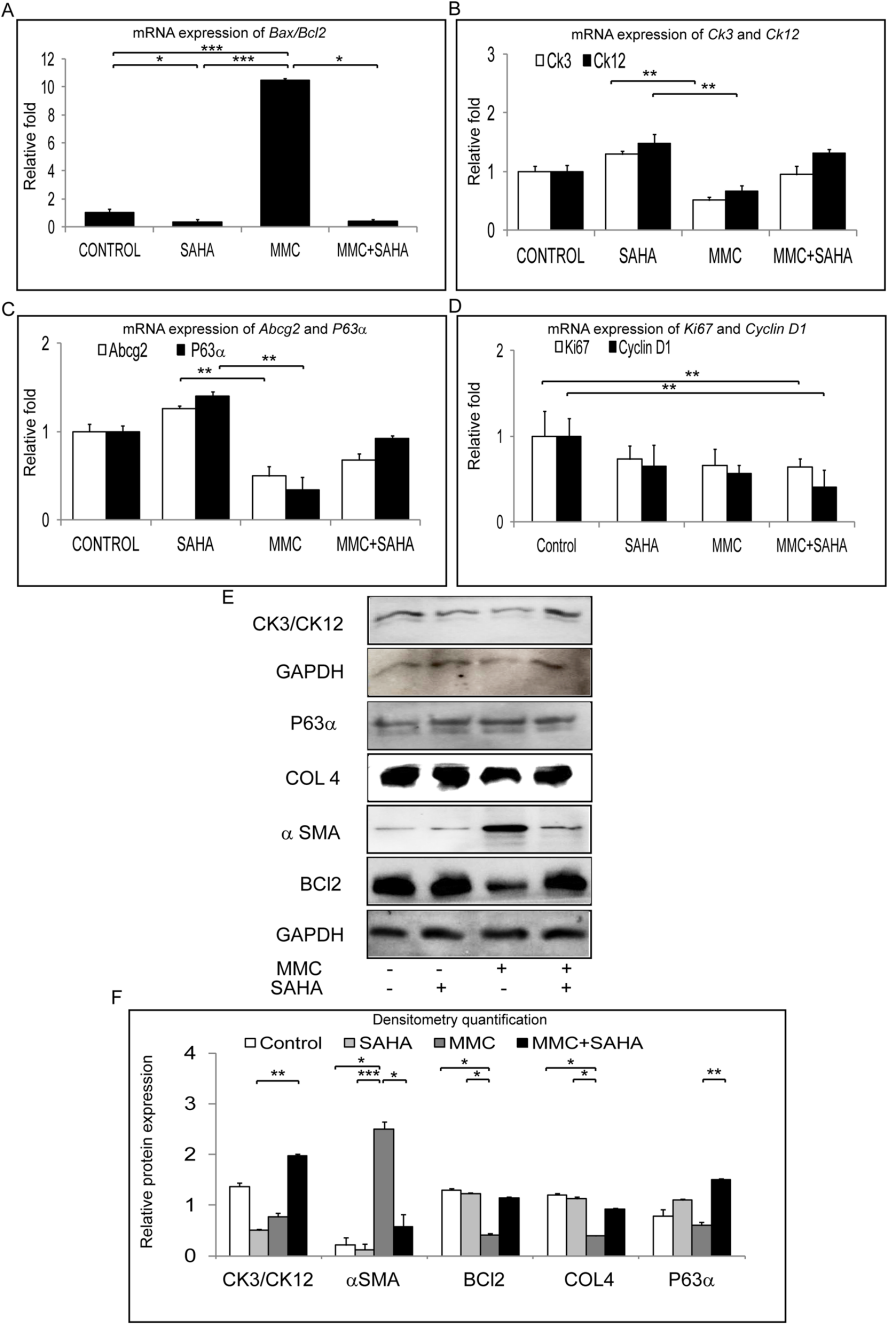
Long term effect of MMC and SAHA on limbal stem/progenitor cells. Ratio of relative mRNA expression of Bax and Bcl2 was estimated in cells obtained from study group 3 (**A**) (n = 4). Relative mRNA expression of Ck3, Ck12 (**B**), Abcg2, ∆NP63 (**C**) and Ki67, Cyclin D1 (**D**) in cells obtained from study group 3 (n = 4). Western blot results showing the expression of CK3/CK12, ∆NP63, COLL4, αSMA, BCl2 and GAPDH in cells obtained from study group 3 (**E**). Graphical representation of relative protein expression quantification of Western blots (n = 3) (F). Statistical significance based on one way ANOVA test denoted by *p ≤ 0.05, **p ≤ 0.01, ***p ≤ 0.001 was calculated. Western blots images were quantified using Image J 1.48 version software (http://imagej.nih.gov/ij/) and statistical analysis performed using statistical software GraphPad PRISM Ver 6.01.

Samples of study group 3 were analyzed in order to evaluate the differentiation potential of the cells in the corneal rim exposed to the different treatments. Phase contrast images revealed decreased number of cultured cells in corneal rims treated with MMC whereas no difference was noted in the cells treated with SAHA, SAHA + MMC and control rims (Supplementary Fig. S1). There was a significantly elevated ratio of Bax to Bcl2 mRNA levels in day 14 differentiated cells obtained from corneal rims treated with MMC (p = 0.0004) compared to control rims. Day 14 differentiated cells obtained from SAHA treated corneal rims, revealed significantly low ratio of Bax to Bcl2 mRNA compared to differentiated cells obtained from untreated control rims (p = 0.0453) and MMC treated rims (p = 0.0004). Day 14 differentiated cells obtained from MMC + SAHA (p = 0.0453) treated corneal rims showed significantly low levels of ratio of Bax to Bcl2 compared to differentiated cells from MMC treated rims (Fig. 7A). The results show that day 14 differentiated cells obtained from corneal rims treated with MMC showed lower mRNA expression of Ck3/Ck12 compared to cultures of SAHA treated corneal rims (p = 0.0022; p = 0.0022). The mRNA levels Ck3 and Ck12 of day 14 limbal epithelial cultures from MMC treated corneal rims were lower than the cells cultured from MMC + SAHA treated corneal rims. mRNA expression levels of Ck3/Ck12 were comparable in corneal rims treated with SAHA alone, SAHA + MMC or control cultures (Fig. 7B). Further, the mRNA expression of corneal epithelial stem/progenitor marker (Abcg2 and ∆Np63) levels of corneal rim treated with MMC were significantly lower than those treated with SAHA (p = 0.0022; p = 0.0022). However, the mRNA expression of Abcg2 and ∆Np63 was higher in day 14 cultured cells obtained from SAHA + MMC treated corneal rims compared day 14 cultured cells obtained from MMC treated corneal rims. The mRNA expression levels of Abcg2 and ∆Np63 were similar in control corneal rims and those treated with MMC + SAHA (Fig. 7C). There was a decrease in the mRNA levels of Ki67 and Cyclin D1 in day 14 differentiated cells obtained from corneal rims treated with SAHA + MMC (p = 0.0022; p = 0.0022) compared to control levels (Fig. 7D). Western blot analysis revealed significantly lower expression of CK3/CK12 in day 14 cultured cells obtained from corneal rims treated with MMC (p = 0.0022) compared to untreated rims. However there was no difference in the expression of CK3/CK12 in day 14 cultured cells obtained from corneal rims incubated with MMC + SAHA and untreated rims. Expression of αSMA was significantly higher in day 14 cultured cells obtained from rims treated with MMC compared to cultured cells obtained from control (p = 0.0453), SAHA (p = 0.0004) and SAHA + MMC (p = 0.0453) corneal rims. No significant difference could be observed in day 14 cultured cells obtained from corneal rims treated with SAHA, MMC + SAHA and controls. There was a significant decrease in the expression of BCL2 in comparison to day 14 cultured cells obtained from rims treated with SAHA (p = 0.0359) and control cultures (p = 0.0254). A significant upregulation in the expression of ∆NP63 levels in day 14 cultures obtained from MMC + SAHA (p = 0.0022) treated rims compared to cultures from MMC treated rims (Fig. 7E,F, Supplementary Fig. S3).

## Discussion

Corneal haze formation post PRK has been one of the primary complications of this laser induced refractive correction procedure. Photorefractive keratectomy (PRK) and laser assisted subepithelial keratomileusis (LASEK) induce corneal epithelial proliferation, migration, differentiation and hemidesmosome formation in an effort to heal the surgically induced epithelial defects^21,22^. Corneal epithelial change drive haze in post-refractive sugery espite the effect in stromal epithelium in development and propagation of sub-epithelial stromal haze^23^. The wound contraction is driven by components of extra-cellular matrix and differentiation of fibroblasts to myofibroblasts or activated keratocytes^24^. Damage to the basement membrane lead to secretion of abnormal stromal extra-cellular matrix components and deposition of abnormal collagen fibrils by activated keratocytes in sub-epithelial regions leading to the formation of haze^25^. The anti-scarring effects are dependent on the keratocyte death^6,26^. The proliferation and migration of the activated keratocytes in the stromal region gets initiated 12–14 h post corneal epithelial injury activated by surgery^6^. However, studies have shown that the status of corneal epithelial cells also contributes in triggering the cascade leading to haze resolution^9^. Further the effect of drugs to treat haze on limbus need to be addressed as long term outcome may lead to limbal stem cell deficiency. Hence, in this study we focused on corneal epithelium and limbal epithelial cells of ex vivo human samples.

It has been shown that a topical application of MMC can lead to an altered phenotype for the corneal epithelial cells as well as a gene expression pattern, as an indicator for cell senescence^27^. The effect of MMC on corneal epithelial cells, keratocytes and corneal endothelial cells is already well known^28^. Animal studies have shown protective effect of SAHA on endothelial cells^26^. It has been illustrated that senescence associated secretory proteins from MMC treated corneal epithelial cells play pivotal role in mitigating the role of MMC in preventing haze^9^. Usage of MMC in ophthalmic surgery was first done as an adjunct in pterygium excision in 1963 and it first use as a corneal healing modulator was performed after almost after more than 35 years in PRK^29,30^. MMC prevents sub-epithelial haze by inducing senescence in activated keratocytes thereby reducing the abnormal ECM by attenuating the TGFβ1 signalling in activated fibroblasts^8,31^. Epithelial basement membrane proteins such as laminin332, perlecan, nidogen are enhanced by MMC treated corneal epithelial cells^9,32^. These proteins play vital role in preventing haze. The cause of haze is being triggered by the initial surgery induced insult to corneal epithelial cells. However, further studies have revealed that MMC reduces corneal fibrosis by reducing collagen deposition and expression of αSMA and fibronectin by corneal fibroblasts and simultaneous deposition of epithelial basement membrane protein Laminin332^9^.

The corneal scarring is induced by excessive wound healing caused by a variety of mechanisms typically involving the TGFβ pathway and its associated proteins. While MMC is clinically used to reduce scarring, its mode of action is to induce apoptosis and inhibition of mitosis in myofibroblast precursors by causing cellular DNA damage^33^. However, in the present study we have used sub-lethal dosage of MMC on in order for us to compare the effects of MMC and SAHA on the differentiated limbal epithelial cells without inflicting any wound. Limbal cells are exquisitely more susceptible to stress compared to corneal fibroblasts and hence clinical application of these drugs needs to be learned.

The effect of MMC treatment on expression of inducible genes can be evaluated if MMC is used at the time of injury but not after wound repair has started. Hartnick et al., found that MMC had no beneficial effect in comparison to placebo in patients undergoing stent or endotracheal tube removal with 1 year follow-up^34^. Similarly our present study is done by treating corneal epithelial cells obtained by differentiating limbal epithelial cells with MMC without any inflicted wound. While this experimental design may be considered a limitation of our study, it should be noted that our aim was to investigate the safety and efficacy of SAHA at the human cornea keeping MMC treatment as a control. MMC is known to have clear negative effects on corneal cells stability and repopulation post refractive surgery, thereby making it important to have alternatives^35,36^. Upregulation of profibrotic genes such as LOX, TGFβ and corresponding decrease in decorin expression demonstrates the pleiotropic effects of MMC in this model of limbal derived epithelial cells.

Decorin, has anti-fibrotic properties and studies have shown its antagonistic functionality towards TGFβ thereby reducing fibrosis^39^. Collagen III, a key component of extracellular matrix along with Collagen I, V, lumican, keratocan and is expressed weakly in cornea in physiological conditions. Its expression increases in a time-dependent manner during wound healing and inflammation^40^. In our experiments cells were cultured in physiological conditions before incubating with MMC, this might have resulted in lower mRNA levels of Col 3A1 gene. It has also been shown that Col 3A1 expression increases in the initial stages of wound healing and eventually replaced with Col 1^41,42^. Col 4A is primarily present in the corneal basement membrane, Bowman’s membrane and Descemet’s membrane with role in development, maintainence and wound healing process of the cornea^43,44^. Treated with TGFβ cells are known to upregulate the expression of myofibroblast markers along with Col 4A^45^. The derranged gene expression profile could be attributed to the DNA interstrand crosslinks that could not be repaired. It has been shown that terminally differentiated cells such as muscle and nerve cells lack a normal DNA repair mechanism resulting in accumulated DNA damage^46^. A similar effect was observed in our differentiated corneal epithelial cultures treated with MMC.

At present MMC remains the primary drug of choice to manage the burden of adverse effects. Reports have shown that prophylactic usage of MMC resulted in poor differentiation of epithelial cells, slow wound healing due to its effect on keratocytes in the stroma, endothelial damage, corneal perforation, limbal and scleral necrosis^37,47,48^. Since, MMC acts on dividing cells, its effects can have long term ramifications^49^. The primary side effect of MMC treatment is endothelial cells damage though there has been no case of endothelial decompensation in any patient^50,51^. However, animal studies of intact goat globe or rabbit model has shown corneal edema and a decrease in endothelial cell density^33,52^. This would prompt an investigation for endothelial cell physiological function on a long term study with MMC. An intact and healthy corneal epithelial cell layer would protect the corneal endothelial cell layer^53^, hence the study was attempted to investigate the role of SAHA in protecting MMC treated corneal epithelial cells. SAHA has been investigated as a plausible alternative to MMC application to avoid haze post refractive surgery. Animal experiments have provided safety and efficacy of SAHA in order to avoid post-refractive laser surgery^18–20^. In continuation with earlier studies, we have evaluated the safety and efficacy of a combinatorial treatment of SAHA and MMC on primary donor derived human corneal cells and tissues.

Our data shows significant decrease in the cell viability at 25 μm concentration of SAHA (Fig. 1). Hence we used 5 μm concentration of SAHA in subsequent experiments. Gronkiewicz et al., have used 2.5 μm of SAHA on canine corneal fibroblast cultures to study its role in corneal fibrosis^54^. Buss et al., have shown that αSMA expression is significantly higher in cultured equine corneal myofibroblasts compared corneal keratocytes and fibroblasts^55^. Interestingly, in our experiments on human donor derived tissues, the expression of αSMA post refractive surgical tissue ablation was significantly reduced by SAHA in comparison to MMC or control treatments. Though MMC has been shown to prevent haze formation successfully, it can induce DNA (CpG-rich promoter, exon, and gene upstream regions) damage leading to abnormal proliferation and gene expression patterns^37,56,57^. Jester et al. using rabbit model noted that higher concentrations of MMC enhanced αSMA in TGFβ-induced corneal keratocytes. They suggested that the increasing expression of αSMA in the presence of MMC is an outcome of DNA damage leading to altered gene expressions^37^. Occleston et al. using Tenon capsule fibroblast cultures have revealed that MMC induced growth arrested cultures are capable of producing growth factors and ECM molecules thereby directly and indirectly affecting the haze either themselves or by altering the behaviour of other cells^58^. Seet et al. showed that MMC treated human Tenon’s fibroblast increased the mRNA expression levels of αSMA and fibronectin. Similar to Occleston et al. report, Seet et al. also noted unperturbed cell migratory property of MMC treated human Tenon’s fibroblast cultures^59^. The increase of αSMA in our study might imply damage in the DNA strand caused by MMC treatment. Moreover, these could be the reason for a the development of haze inspite of MMC treatment^59,60^. Abnormal proliferation and gene expression pattern of cells have been implicated to the DNA damage induced by MMC^37,61^. MMC is known to have toxic effects on corneal epithelial cells^35^. Hence, we used 0.005% MMC in our study. Gupta et al. have a shown that a single 0.02% of MMC was not toxic for canine keratocyte and fibroblast cultures^62^. Jester et al. showed that 0.02% of MMC was inducing cell death in cultured rabbit keratocytes and lower concentrations were blocking or reducing cell proliferation^61^. Corneal stromal lenticules treated with SAHA demonstrated reduced expression of αSMA with 1 week as well as 1 month exposure. Tandon et al. have shown in rabbit and human corneal fibroblasts that SAHA reduced the expression of αSMA^26^. In the presence of TGFβ stimulation on corneal lenticules, SAHA prevented the mRNA expression of αSMA, as described in other systems^63^.

Clinically effective dosage of MMC is 0.02–0.4 mg/ml (0.002–0.04%) used intraoperatively^64,65^. Subconjunctival injection of SAHA (9.25 μg/ml and 50 μM) were given in New Zealand white rabbits to improve bleb survival and prevent fibrosis in Glaucoma filteration surgery^66,67^. Topically 25–350μM of SAHA has been administered in New Zealand white rabbits and Sprague–Dawley rats respectively, to prevent corneal haze/scarring post PRK^20,26,68^. However, in monolayer cultures concentrations ranging from 5 to 55 μM has been used to prevent corneal fibrosis^68,69^.

Cell viability assays revealed that MMC treated 14-day limbal epithelial cultures as well as corneal rims had significantly lower percentage of viable cells in agreement with other studies which have shown MMC induced cell apoptosis^70^. This observation is further supported by the enhanced ratio of mRNA levels of Bax/Bcl2. We as well as others have a shown that the ratio of Bax/Bcl2 determines the cell susceptibility to apoptosis^71–74^. However, in the presence of SAHA, the MMC induced apoptosis of cells were prevented. Woo et al., showed higher TUNEL-positivity implicating higher apoptosis in rat cornea on MMC treatment was significantly reduced when low dosage of MMC treatment was combined with SAHA^68^. Kim et al., also showed that a low dose of MMC along with SAHA prevented apoptosis of conjunctival epithelial cells as well as tenon capsule fibroblasts in rabbits^75^. Cells obtained from corneal rims exposed to MMC showed significantly lower BCL2 positivity compared to controls. Thus, the data demonstrates that in the presence of SAHA, deleterious effect of MMC is mitigated.

MMC, SAHA and their combination treatment decreased the expression of corneal fibrotic markers (TGFβ, LOX and COL4A) as shown by the mRNA as well as immunofluorescence results. Similarly, Anumanthan et al., have shown using rabbit models of corneal haze that MMC and SAHA are effective in preventing corneal haze post photorefractive keratectomy^20^. Woo et al. have shown using rat models and cultured human corneal epithelial cells that though MMC and SAHA prevent proliferation of corneal myfibroblasts, a combination of low dosage of MMC with SAHA is better as MMC at high dosage is toxic to cells^68^. Conjunctival epithelial cells cultured from rabbits were used to study subconjunctival fibroblast in the presence of MMC and SAHA. Expression of proliferative markers was also reduced in the presence of MMC, SAHA and a combination of MMC and SAHA. Studies have reported the role of HDAC inhibitors in preventing cellular proliferation^76^. Additionally, the MMC is known to prevent proliferation of keratocytes^20,68^. Our findings are in agreement with the known mechanism that suppression of myofibroblast proliferation would prevent fibrosis formation^68^. Hence, a combination of MMC + SAHA might have better clinical applicability in blocking corneal haze post PRK.

SAHA is a known modulator of MDR group of proteins^77^. Hence, our results revealed that in the presence of SAHA and MMC + SAHA there was an upregulation of the mRNA/protein expression of Abcg2 and Abcb1 both in cells obtained from corneal rims as well as in cultured differentiated limbal epithelial cells. It has been shown that through the activation of MDR proteins effluxes MMC thereby reducing the harmful effects of MMC on cells^78,79^. It has been shown that Abcb1 and Abcg2 play crucial role in imparting efflux of MMC^80,81^.

Mitomycin C belongs to a class of antitumor antibiotics that needs to undergo bioreductive alkylation by enzyme systems such as DT-diaphorase, NADPH-cytochorme P-450 reductase, NADPH-cytochorme C reductase, xanthine oxidase and falvoprotein transhydrogenase^82^. The reductive activation results in semiquinone or hydroquinone metabolite that has cytotoxic capability^82,83^. The alkylating groups of MMC bind to two nitrogen atoms forming interstrand DNA links thereby crosslinking the DNA strands and inhibiting cell proliferation^84^. MMC has been primarily used in cancer treatment primarily because of its antiproliferative role^82^. Intracellular accumulation of MMC results in high number of DNA crosslinking leading DNA damage. This eventually prevents normal DNA replication causing cell death^85–88^. It has been shown that low dosage of MMC activated Abcb1 gene and protein^89,90^. It has been shown that COX-2 inhibitors prevent induction of MDR proteins^86^. Co-administration of COX2 inhibitor, celecoxib with MMC in COX2- deficient urinary bladder cell line UMUC-3, resulted in increased intracellular MMC levels^86^. The cytosolic concentration of MMC has been reported in the literature^85–88^. Wilson et al. using colon cancer cell line showed that resistance to MMC was induced by decrease in DNA crosslink formation^91^. Resistance to MMC is mediated partly by blocking the alkylation of MMC and also by efflux mechanism of multi-drug resistance proteins^92,93^. Dorr et al. using a mouse leukaemia cell line, L1210 demonstrated that resistance to MMC is imparted by expression of *P*-glycoprotein on the membrane and decreased accumulation of intracellular MMC^94^. In similar most likely, our results here support the efflux mechanism of imparting MMC resistance, since SAHA induces expression of *P*-glycoprotein as well as BCRP, multi-drug resistant proteins.

The safety of SAHA and MMC treatments were evaluated in a longer timescale by culturing the donor corneal rim derived primary cells. These cells were cultured in corneal differentiation medium to evaluate the effect of treatment on cell differentiation and proliferation capacity. The corneal rim harbours limbal epithelial as well as transient amplifying cells which are essential for the health of the corneal surface^95^. Results revealed that a combinatorial treatment with SAHA and MMC prevented any compromise of the differentiation potential towards corneal lineage cells when compared to MMC alone. Moreover, the rims exposed to MMC + SAHA did not show any adverse effects on corneal stem/progenitor marker population when compared with MMC treatment.

These findings implicate that a combination treatment of MMC + SAHA or possibly SAHA alone, could effectively prevent corneal haze formation post PRK surgery in human eyes by reducing fibrosis without excessive cell death or compromised corneal cell differentiation. Additionally, our in-vitro data suggests that the combination treatment of MMC + SAHA might also be safe in the long term with respect to maintaining cellular health. Further clinical trials with a combinatorial treatment on post PRK complications are warranted to establish this treatment as a standard of care to prevent corneal haze.

## Materials and methods

### Human corneoscleral rim collection and limbal primary culture

The current study was approved by the Narayana Nethralaya institutional Review Board. Samples were collected as per Narayana Nethralaya ethics committee (Bangalore, Karnataka, India) and adhered to the tenets of the Declaration of Helsinki guidelines. Informed written consent was obtained from all subjects prior to sample collection. The tissue used for this study was sourced from the Shankar Anand Singh Eye Bank (Bangalore, Karnataka, India). The residual corneoscleral rim post corneal transplantation was used for the limbal primary culture. Tissue samples were collected from subjects within the age group of 25–65 years and both genders were included. We used 50 corneoscleral rims in this study. Limbal primary culture was carried out with modification of a previously described protocol^95^. Briefly, the excessive sclera and peripheral cornea were trimmed from the corneal rim. The enzymatically treated Dispase II (2 mg/ml in DMEM; Sigma-Aldrich Missouri, USA) for 30 min at 37 °C, 5% CO_2_) corneoscleral rim was then chopped into small pieces, placed on de-epithelialized human amniotic membrane (dHAM) and cultured for obtaining corneal epithelial cells using growth medium. The growth medium contained Dulbecco’s Modified Eagle’s Medium (DMEM)/Ham’s F12 nutrient mix (v/v, 1:1), human recombinant EGF (10 ng/ml), human recombinant insulin (5 μg/ml) (Gibco, Grand Island, New York, USA), penicillin (100 U/ml), streptomycin (100 μg/ml) and amphotericin B (2.5 μg/ml) (HiMedia, Mumbai, Maharashtra, India) along with 10% fetal bovine serum (Gibco, Grand Island, New York, USA). The protocol followed for limbal cells culture was the explant culture technique.

#### Study group 1

Cultured limbal epithelial cells differentiated to corneal lineage after 14 days of culture are used. These cells are treated 24 h with 5 µM SAHA, 0.005% MMC, 0.005% MMC + 5 µM SAHA or mock control. These cells are then collected for further analysis for mRNA or protein.

#### Study group 2

In this group corneoscleral rims were treated with 5 µM SAHA, 0.005% MMC, 0.005% MMC + 5 µM SAHA or mock control for 24 h. At the end of incubation, the peripheral cornea and the sclera tissue was trimmed from these corneoscleral rims. Finally, the limbal epithelial cells were scarped and collected for mRNA and protein analysis^95^.

#### Study group 3

In this last group corneoscleral rims were treated with 5 µM SAHA, 0.005% MMC, 0.005% MMC + 5 µM SAHA or mock control for 24 h. At the end of incubation, limbal epithelial cells were isolated from the rims. Cells obtained by the rims were cultured on de-epithelialized human amniotic membrane and differentiated to corneal lineage by growing them for 14 days. At the end of 14 days cells were collected for mRNA and protein analysis^95^.

### Human lenticule and treatment

Lenticules (n = 4) obtained intraoperatively during SMILE surgery from each subject undergoing refractive correction surgery were included in the study. Each lenticule was cut into four equal pieces and were subjected to 48 h TGFβ (10 ng/ml), SAHA (5 µM), combination of SAHA (5 µM) + TGFβ (10 ng/ml) treatment with untreated control in DMEM-F12 media supplemented with 20% FBS, 1% Antibiotic Antimycotic solution. Upon completion of 48 h incubation, total RNA was extracted from each lenticule followed by cDNA conversion and amplified using real time PCR for αsmooth muscle actin (pro-fibrotic marker) and interleukin 6 (pro-inflammatory marker) expression levels. Actin served as housekeeping control.

### Human donor cornea collection and treatment

Donor human corneas unsuitable for transplantation were subjected to excimer laser ablation. Corneal tissues (n = 4) were treated with MMC (0.005% per ml) and SAHA (5 µM) for 1 week and 1 month to study laser induced wound and differential healing response. Post 1 month treatment, corneal button was cut into two halves, one part subjected for gene expression analysis and other half was used for making corneal paraffin blocks. After 1 week treatment, total RNA was isolated from the treated corneal tissues and were analysed for α-smooth muscle actin.

### Cell viability count-Trypan blue

Cell viability of corneal epithelial cells treated with and without SAHA, MMC was accounted by using trypan blue staining. Cells are detached from by using 0.25% trypsin EDTA and collected. Cells are further diluted to a suitable concentration and 0.4% (w/v) trypan blue (Mediatech, Cellgro, USA) is added to the media containing cells. Then the viable and non-viable cells were counted with the aid of haemocytometer and the percentages of viable cells were determined and represented graphically.

### RNA extraction, cDNA conversion and quantitative RT-PCR

Briefly, TRIzol reagent method (Ambion, Carlsbad, CA, USA) was used to extract the total RNA from the cells treated with and without SAHA, MMC, MMC + SAHA, cultured on dHAM. It has been quantified with Nanodrop spectrophotometer 1000 (Thermoscientific, Wilmington, USA) and complementary DNA was generated from those samples using High capacity cDNA Reverse Transcription Kit, (Life Technologies, CA, USA) and stored at − 20 °C. Quantitative real time-PCR was performed by using KAPPA SYBER FAST qPCR Master Mix (Kapa Biosystems, Wilmington, MA, USA), and analyzed on Bio-Rad CFX Connect (Bio-Rad, Hercules, CA, USA) and Quantitative RT-PCR was performed for corneal epithelial differentiation markers (CK3, CK12), epithelial mesenchemal transition markers (αSMA, LOX, TGFβ, and COLL4), apoptosis markers (Bax and Bcl2) and inflammation marker (IL-6) data were normalized with the expression of housekeeping gene GAPDH. All the experiments were done in triplicate. The primers used are listed in Table 1.

**Table 1:** Primers used in RT-qPCR.

Gene primer & Accession no	Forward sequence	Reverse sequence
*gapdh* *NM_001289746.1*	ACCCACTCCTCCACCTTTGAC	TGTTGCTGTAGCCAAATTCGTT
*bax* NM_138761	TTGCTTCAGGGTTTCATCCA	AGACACTCGCTCAGCTTCTTG
*bcl2* NM_ 000633	TGGCCAGGGTCAGAGTTAAA	TGGCCTCTCTTGCGGAGTA
α*SMA* *NM_001141945.1*	GCTGGCATCCATGAAACCAC	TACATAGTGGTGCCCCCTGA
*TGF*β *NM_000660.5*	CAGCAACAATTCCTGGCGATACCTC	CAACCACTGCCGCACAACTC
*Lox* *NM_002317.5*	ACATTCGCTACACAGGACATC	TTCCCACTTCAGAACACCAG
*Col4A1* *NM_001845.5*	GCAAACGCTTACAGCTTTTGG	GGACGGCGTAGGCTTCTTG
*Il6* *NM_000600.3*	GATGAGTACAAAAGTCCTGATCCA	CTGCAGCCACTGGTTCTGT
*Decorin* *NM_ 001920.3*	GCTTCTTATTCGGGTGTGAGT	TTCCGAGTTGAATGGCAGAG
*Fibronectin* *NM_ 212482.1*	TGGCCAGTCCTACAACCAGTA	
*Collagen 3A1* *NM_ 000090.3*		
*abcg2* *NM_004827*	GAGCCTACAACTGGCTTAGACTCA	TGATTGTTCGTCCCTGCTTAGAC
*p63α* *NM_001114980*	AGCCAGAAGAAAGGACAGCA	CAGGTTCGTGTACTGTGGCT
*Ck3* *NM_057088*	AGTTTGCCTCCTTCATTGACA	TGCCTGAGATGGAACTTGTG
*Ck12* *NM_000223*	ACGAGCTGACCCTGACCA	CGGAAGCTTTGGAGCTCAT
*Cyclin D1* *NM_002592*	AGCTCCTGTGCTGCGAAGTGGAAAC	AGTGTTCAATGAAATCGTGCGGGGT
*Ki67* *NM_002417*	CTTTGGGTGCGACTTGACG	GTCGACCCCGCTCCTTTT

### Western blot

Cells were pelleted by centrifugation (Eppendorf, HAM, Germany) and analysed using the RIPA (RadioImmunoPrecipitation Assay) buffer (G-Bioscience, St. Louis, MO, USA). The cell lysates were centrifuged at 12.4 × 1000g for 30 min and the total protein concentration of the supernatants was quantified using the BCA (BicinChonic Acid) assay (Thermo Scientific, Rockford, IL, USA). A 10% sodium dodecyl sulfate–polyacrylamide gel electrophoresis was used to separate proteins and wet-transferred (Rugby, WAR, UK) using polyvinylidene fluoride membrane. Membranes were then blocked and primary antibodies were used with an optimal 1:1000 dilution and incubated overnight. The secondary antibody radish peroxidase conjugates (1:5000; BioLegend, San Diego, CA, USA) incubated 1 h horse. Protein bands were developed using an enhanced chemiluminescence detection kit (Pierce ECL Plus; Thermo Scientific) and analyzed with the Image Quant LAS 500 chemiluminescence detector (GE Healthcare Life Science, Uppsala, Sweden). Primary and secondary antibodies are listed in Table 2.

**Table 2:** Details of antibodies used in immunofluorescence.

Primary antibodies	Dilution	Vender
αSMA, mouse	1:50 (IF); 1:1000 (WB)	Abcam, USA
TGFβ, rabbit	1:50 (IF)	Santacruz Biotechnology, USA
Collagen 4, rabbit	1:50 (IF): 1:1000 (WB)	Abcam, USA
BCL2, rabbit	1:250 (IF); 1:1000 (WB)	Abgenex, India
CK3/12, rabbit	1:1000 (WB)	Abcam, USA
ABCG2-APC, mouse	1:20 (FACS)	Biolegend, USA
APC-IgG2b, κ isotype, mouse	1:20 (FACS)	Biolegend, USA
P63α, rabbit	1:1000 (WB)	Cell Signaling, USA
GAPDH, mouse	1:1000 (WB)	Abgenex, India
**Secondary antibodies**		
Donkey anti-rabbit IgG, Cy3	1:500	Jackson Immunoresearch, USA
Donkey anti-rabbit IgG, Alexa flour 488	1:500	Abgenex, India
Donkey anti-mouse IgG, Cy3	1:500	Jackson Immunoresearch, USA
Donkey anti-rabbit IgG, HRP	1:4000	Biolegend, USA
Goat anti-mouse IgG, HRP	1:4000	Biolegend, USA

### Fluorescent activated cell sorting (FACS) analysis

Limbal epithelial stem cells differentiated to corneal epithelial cells treated with and without SAHA, MMC, MMC + SAHA for 24 h and untreated cells cultured on dHAM. These cells were stained for ABCG2-APC (MDR protein) (BioLegend, San Diego, CA, USA). Cells were stained for FACS analysis. Briefly, cells were trypsinized and fixed with 4% paraformaldehyde before being permeabilized with 0.1% Triton X (Qualigens, Mumbai, India). The permeablized cells were stained with antibody for ABCG2. Unstained cells and cells stained with secondary antibodies alone were used as controls. The fluorescence emitted by cells in FL2 channel was recorded and analyzed using BD CellQuest Pro software, (BD Biosciences, CA, USA)^96^. Primary and secondary antibodies are listed in Table 2.

### Immunofluorescence

Scrapped cells obtained from treated corneal rims were fixed using 2% paraformaldehyde (Sigma Aldrich, MO, USA) for 10 min, permeabilized with 0.1% Triton X 100 (Thermo Fischer Scientific, Mumbai, India) in 1× PBS for 15 min, blocked with 1% bovine serum albumin/phosphate-buffered saline (Himedia, Mumbai, India) at room temperature for 1 h. The blocked samples were incubated with primary antibody overnight at 4 °C. After a brief rinse in 1× PBST (0.02% Tween 20 (MP Biomedicals, CA, USA), cells were incubated with secondary antibodies for 1 h. The slides were finally mounted using Vector shield containing 2-(4-amidinophenyl)-1H-indole-6-carboxamidine (DAPI) aqueous mounting medium (Vector laboratories, CA, USA). The fluorescent images were documented using ProgRes Capture Pro 2.5 software on fluorescent microscope (Olympus BX41). Fluorescence intensity was quantified using Image J 1.48 version software (http://imagej.nih.gov/ij/; provided in the public domain by the National Institutes of Health, Bethesda, MD, USA)^74^. Primary and secondary antibodies are listed in Table 2.

### Immunofluorescence of corneal tissues

4 µm sections of MMC and SAHA treated corneal tissues were deparaffinised, rehydrated, and heated in antigen retrieval citrate buffer (pH 6) solution. Sections were incubated with blocking buffer (3%BSA in 1 × PBS) for 1 h at room temperature. Slides were then incubated overnight with mouse monoclonal anti α-smooth muscle actin (Abcam, ab7817; dilution 1:500) followed by secondary antibody Anti-mouse Cy3 (715–165–150, Jackson ImmunoResearch, dilution 1:2000). Further 4,6-diamidino-2-phenylindole (DAPI) staining, slides were mounted in Fluoroshield (Sigma) and stored at 4 °C in the dark. Microphotographs were obtained on Olympus CKX53 inverted microscope. Primary and secondary antibodies are listed in Table 2.

### Statistical analysis

All the experiments were performed in triplicate and results of three independent experiments were used for statistical analysis. Data are represented as the mean ± SD and were analyzed with one-way ANOVA and Mann–Whitney U test for multiple group comparison using statistical software GraphPad PRISM Ver 6.01. Significance value denoted, p* < 0.05, ** < 0.01, *** < 0.001, **** < 0.0001.

## Supplementary Information

Supplementary Information
