# Supplementary material for: Safety and efficacy of combination of suberoylamilide hydroxyamic acid and mitomycin C in reducing pro-fibrotic changes in human corneal epithelial cells
Source: Sci Rep. 2021 Feb 23;11:4392. doi: 10.1038/s41598-021-83881-y (PMC7902619; doi:10.1038/s41598-021-83881-y)
Supplement: Supplementary file 1 — Supplementary Information [file 41598_2021_83881_MOESM1_ESM.pdf]

# Safety and efficacy of combination of Suberoylamilide Hydroxyamic Acid and Mitomycin C in reducing pro-fibrotic changes in human corneal epithelial cells


Rohit Shetty[1], Nimisha R Kumar[2], Murali Subramani[3], Lekshmi Krishna[3], Ponnalagu Murugeswari [3], Himanshu Matalia[1], Pooja Khamar[1], Zelda V Dadachanji[1], Rajiv R Mohan[4,5,6], Arkasubhra Ghosh[2*], Debashish Das[3*]

[1]Department of Cornea and Refractive Surgery, Narayana Nethralaya Eye Hospital, Bangalore, Karnataka, India

[2]GROW Laboratory, Narayana Nethralaya Foundation, Narayana Nethralaya, Bangalore, Karnataka, India.

[3]Stem Cell Research Lab, GROW Laboratory, Narayana Nethralaya Foundation, Narayana Nethralaya, Bangalore, Karnataka, India.

[4]Department of Veterinary Medicine and Surgery, University of Missouri, Columbia, MO, 65211, USA.

[5]Mason Eye Institute, School of Medicine, University of Missouri, Columbia, MO, 65212, USA.

[6]Harry S Truman Veterans' Memorial Hospital, Columbia, MO, 65201, USA.

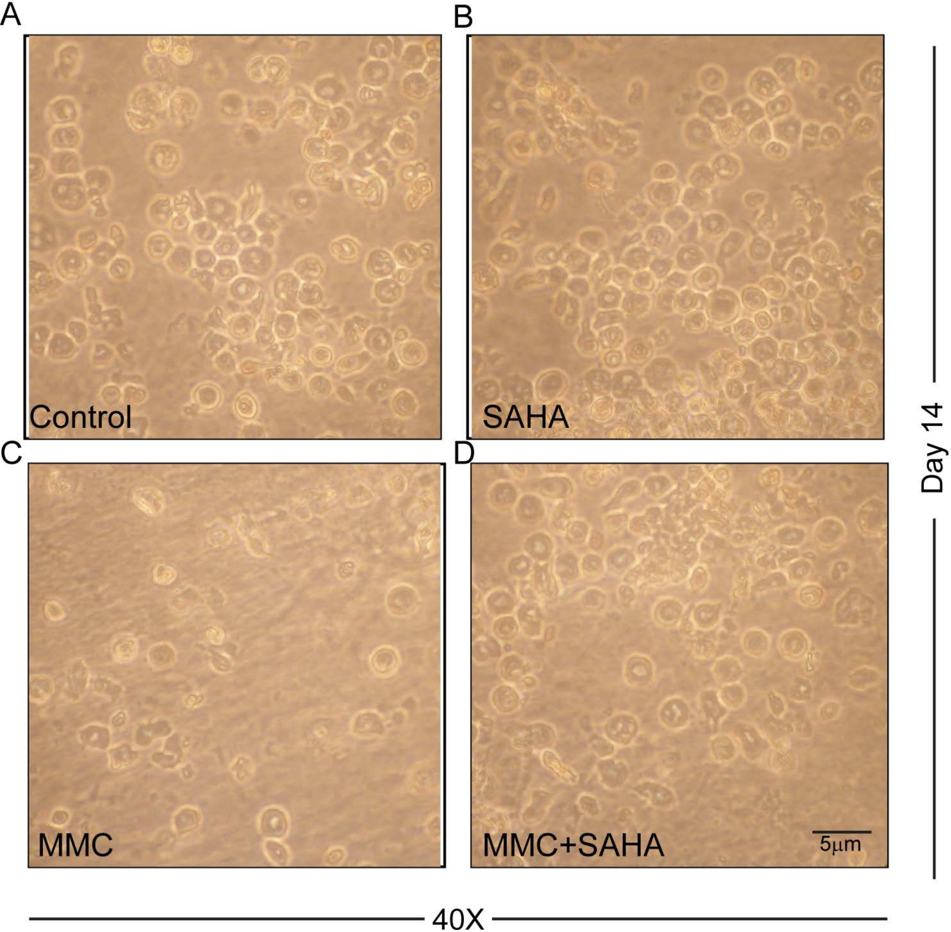


Supplementary Figure 1

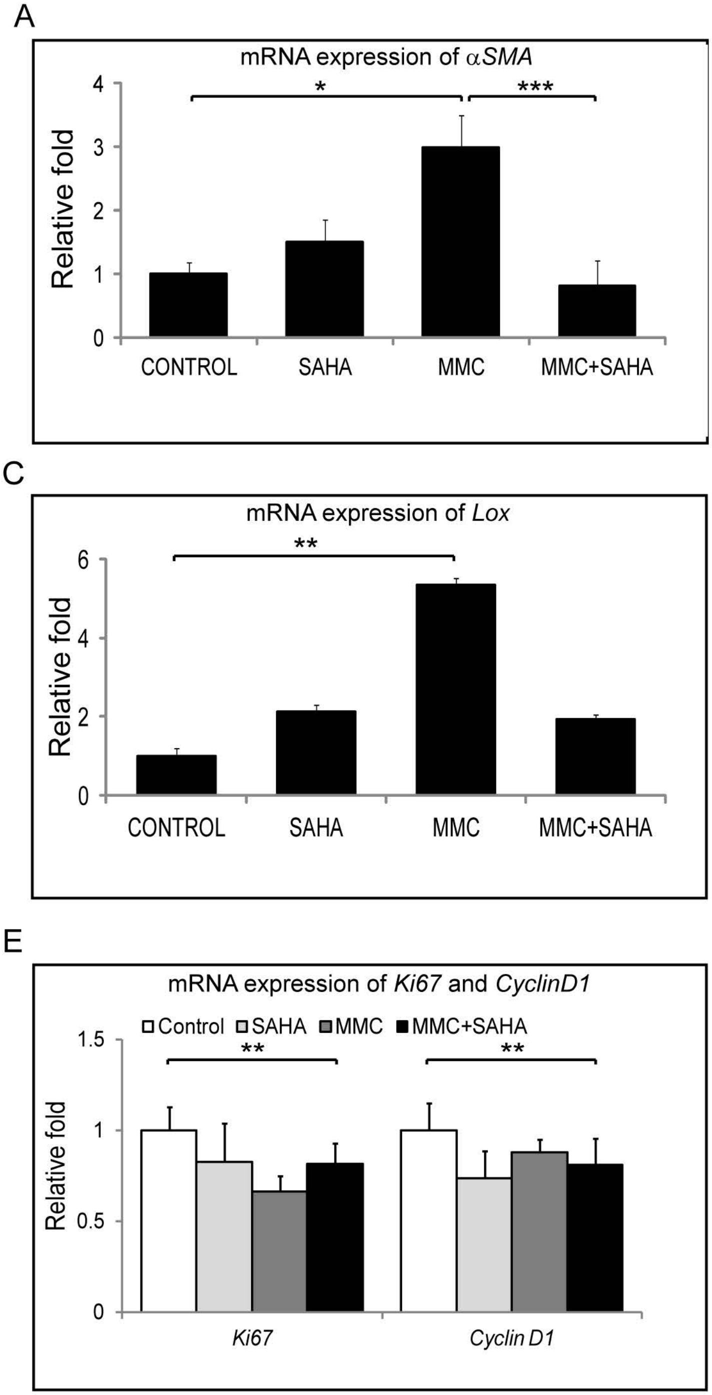


Supplementary Figure 2

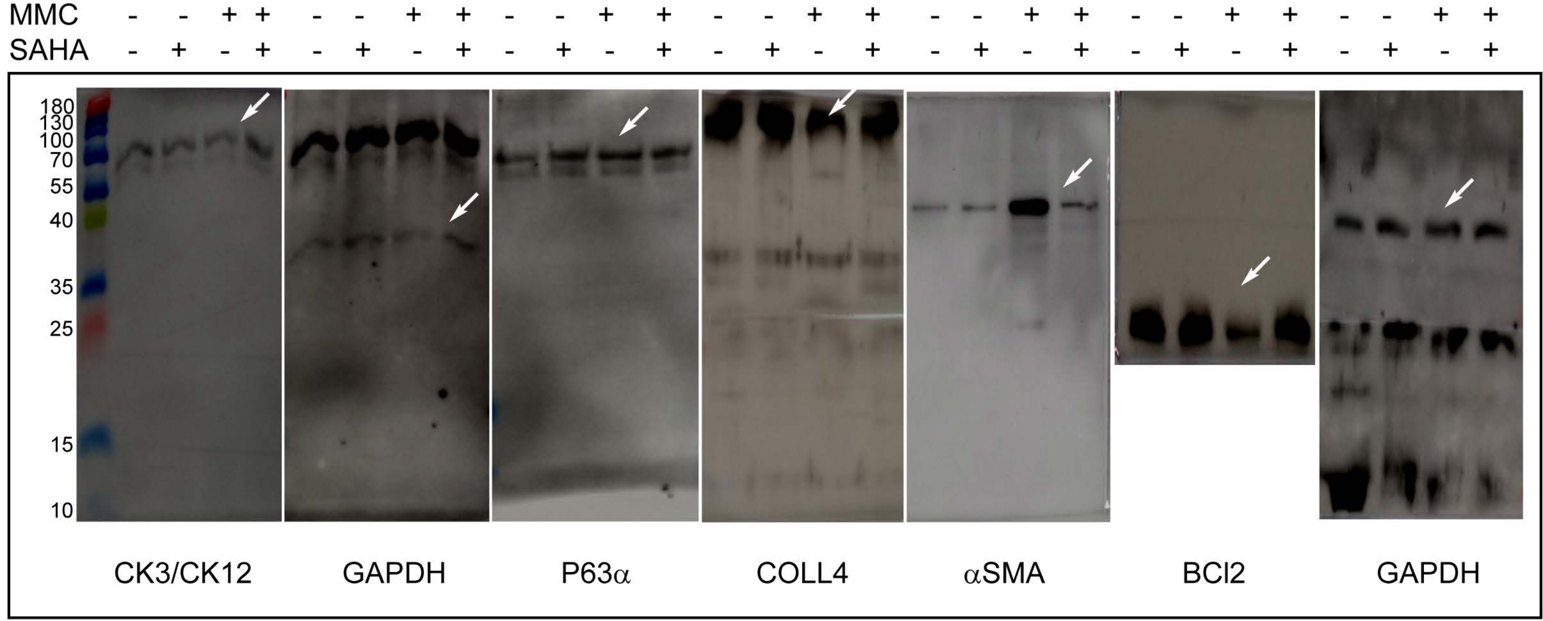


Supplementary figure 3
